# Fibrous Dysplasia of the Ethmoid Bone Diagnosed in a 10-Year-Old Patient

**DOI:** 10.3390/medicina61010045

**Published:** 2024-12-31

**Authors:** Zofia Resler, Monika Morawska-Kochman, Katarzyna Resler, Tomasz Zatoński

**Affiliations:** 1 Student Scientific Society of Otolaryngology, Department of Otolaryngology, Head and Neck Surgery, Wroclaw Medical University, 50-556 Wrocław, Poland; 2 Department of Otolaryngology, Head and Neck Surgery, Wroclaw Medical University, 50-556 Wrocław, Poland; monika.morawska-kochman@umw.edu.pl (M.M.-K.); tomasz.zatonski@umw.edu.pl (T.Z.)

**Keywords:** fibrous dysplasia, children, bone disease, ethmoid bone, osteitis, craniofacial lesion

## Abstract

Fibrous dysplasia is an uncommon bone disorder affecting various parts of the skeleton, often affecting facial and cranial bones. In this case, a 10-year-old patient was diagnosed with fibrous dysplasia of the ethmoid sinus at an early age. The patient has experienced nasal congestion, snores, and worsening nasal patency since 2019. A CT scan revealed an expansive proliferative lesion, likely from the frontal or ethmoid bone, protruding into the nasal cavity, ethmoid sinus, and right orbit. The tumor causes bone defects in the area of the nasal bone, leading to fluid retention in the peripheral parts of the right maxillary sinus. The patient’s parents decided not to undergo surgery to remove the diseased tissue and reconstruct the area, as it would be very extensive, risky, and disfiguring. The patient is being treated conservatively with an MRI, with a contrast performed approximately every six months and infusions of bisphosphonates. Despite the lesion’s size, the patient does not experience pain characteristic of dysplasia, and functions typically. Fibrous dysplasia of bone is a rare condition that presents with the most visually apparent manifestations, often mistaken for other bone conditions. Advanced diagnostic tools, like CT and MRI, are used to identify conditions affecting the ethmoid sinus more frequently. However, diagnostic errors often occur in imaging studies, leading to confusion. The most common period for clinical manifestations and diagnosis is around 10 years of age. The preferred approach in managing fibrous dysplasia involves symptomatic treatment, which can alleviate airway obstruction, restore normal globe position and visual function, and address physical deformities. Surgical intervention is recommended only for patients with severe functional impairment, progressive deformities, or malignant transformation.

## 1. Introduction

**Figure 1:**
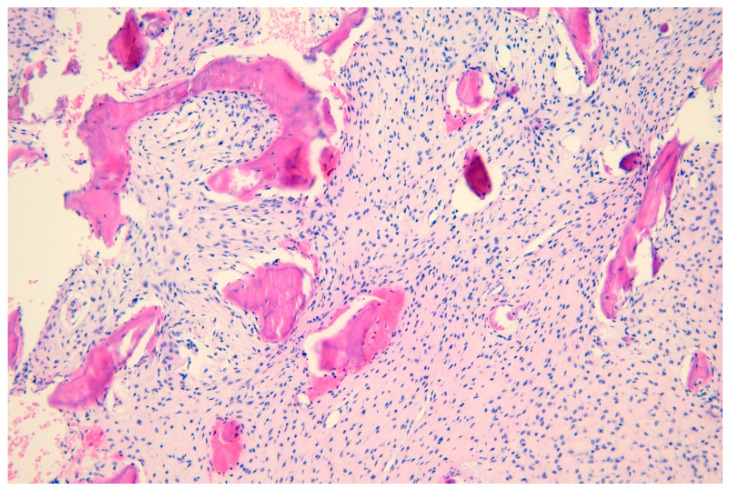
Histopathological findings. Tumor tissue composed of diffuse irregular, circular attenuated bone trabeculae on a background of fibrous tissue (H + E, ×100).

**Figure 2:**
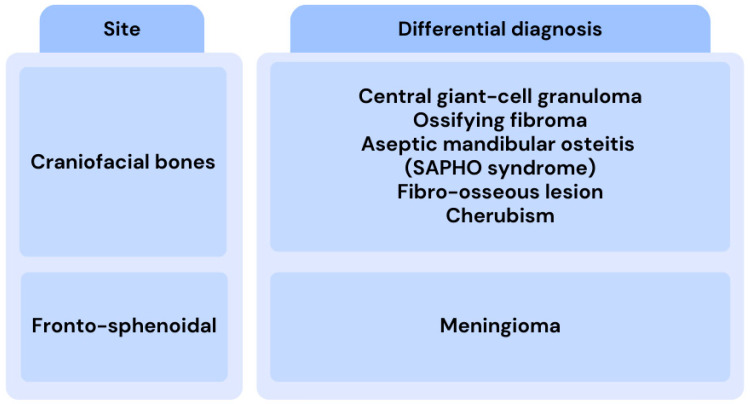
Differential diagnosis of craniofacial fibrous dysplasia [7].

Fibrous dysplasia (FD) is an uncommon bone disorder that can impact various parts of the skeleton, primarily affecting facial and cranial bones, such as the mandible and maxilla [1]. An irregular mix of weak fibrous and osseous tissue replaces the normal bone marrow in the affected bones in fibrous dysplasia. The precise etiology of the disease is currently not fully elucidated [1,2]. However, some studies suggest that fibrous dysplasia is caused by sporadic post-zygotic mutations in the *GNAS* gene located on chromosome 20 [3,4]. The mutation disrupts the intrinsic GTPase activity of *Gsα*, causing receptor activation. This leads to abnormal cyclic adenosine monophosphate (cAMP)-mediated signaling, which results in elevated cAMP levels within mutated cells. Mutated osteoblastic cells overexpress the receptor activator of nuclear factor kappa-B ligand (RANKL) and interleukin-6 (IL-6), which subsequently activate osteoclasts. This activation promotes bone resorption, replacing normal bone with an overproduced, disorganized collagenous matrix, ultimately causing the FD lesion to expand [3,5] (Figure 1). Fibrous dysplasia has a female predilection [6]. This condition has three forms: monostotic (70% of cases), polyostotic (30% of cases), and the most severe form, McCune-Albright syndrome, which is defined as the combination of FD and one or more extra-skeletal features, or the presence of two or more extra-skeletal features, such as skin pigmentation manifestation and premature sexual maturation [7,8]. The monostotic form involves one skeletal site without extra-skeletal manifestations, usually a benign presentation encountered in the second and third decades of life. In contrast, the polyostotic form affects multiple bones without extra-skeletal manifestations [7,9]. It often presents with significant morbidity, thus requiring careful management to alleviate symptoms and prevent complications such as fractures or deformities associated with the condition [10]. Isolated monostotic bone lesions have a wide differential diagnosis and often involve diagnostic uncertainty, usually require histological confirmation. Moreover, in some cases a molecular diagnosis of affected tissues is indicated (Figure 2). Management strategies for fibrous dysplasia typically involve a multidisciplinary approach, including orthopedic intervention, pharmacological treatment for pain relief, and regular monitoring to assess potential complications, such as osteosarcoma, in patients with the polyostotic form of the disease.

Fibrous dysplasia of the ethmoid sinus is rarely encountered, and diagnosing it at such an early age as in our 10-year-old patient is exceptionally uncommon. This case illustrates the challenges of diagnosing fibrous dysplasia, especially in pediatric patients, where clinical manifestations may overlap with other conditions, leading to potential misdiagnosis or delayed treatment [10].

## 2. Case Description

**Figure 3:**
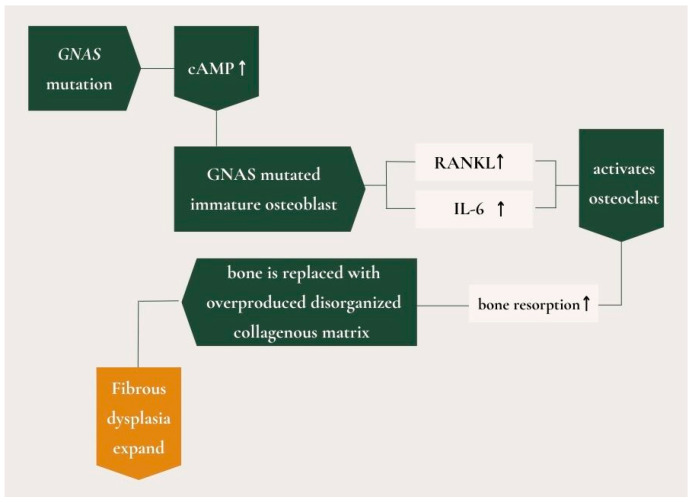
The pattern of cascade of events resulting from GNAS mutations leading to fibrous dysplasia. (↑—rise).

A 10-year-old patient was admitted to the hospital for a scheduled computer tomography (CT) scan of the craniofacial area due to suspected growth changes in the right nasal cavity. During the interview, the patient testified to experiencing nasal congestion for a long time. Since 2019, her symptoms have worsened. The child breathes through the mouth constantly, snores and has experienced a significant worsening of nasal patency. The patient’s mother also reports observing gradual changes in her daughter’s facial features on the right side. The patient states that there have been no previous bleeding episodes or pathological discharge from the nasal cavity. Nasal endoscopy revealed proliferative changes in the right nasal cavity, causing obstruction of the right nasal passage and associated external swelling at the base of the nose. No pathological discharge was observed in the nasal cavities. Ocular examination revealed a mild strabismus of the right eye, with round, normally reactive pupils. A contrast-enhanced CT scan of the craniofacial and neck region was performed for a more detailed diagnosis. The examination revealed an expansive proliferative lesion, most likely originating from the frontal or ethmoid bone and protruding into the nasal cavity, ethmoid sinus, and right orbit. The lesion exhibited a peripheral structure consistent with preserved bone structure, with islands, calcifications, and ossifications mainly localized at the lower pole of the tumor. The central part of the lesion was filled with irregular space containing dense fluid. The overall dimensions of the lesion were 35 mm in transverse diameter, 43 mm in anteroposterior diameter, and 52 mm in craniocaudal diameter. The tumor causes the formation of bone defects in the area of the nasal bone. It leads to fluid retention in the peripheral parts of the right maxillary sinus. Additionally, it affects the right nasal conchae and the palate. It causes exophthalmos and the peripheral displacement of the right eyeball. The remaining facial and temporal bones exhibit no structural changes. The anatomical spaces of the face and neck present a picture within normal limits. In the CT scan examination, the image of the lesion was not conclusive (Figure 3). Initially, fibrous dysplasia was considered for differentiation from osteofibrous dysplasia as a possible diagnosis. The patient was subsequently scheduled for a biopsy of the lesion under general anesthesia. After preparing the surgical field under endoscopic visualization, the mucosa was anesthetized and incised, revealing a significant protrusion of the sphenoid bone. The histopathological examination revealed a sample composed of bony trabeculae with typical architecture. The intertrabecular spaces were filled with bone marrow tissue and predominantly vascular fibrous connective tissue (Figure 1). In April 2020, the patient underwent a significant resection of the lesion in a part of the nasal cavity. Unfortunately, the resection did not bring any positive changes. An MRI performed in August 2020 showed a lesion of dimensions similar to those before the biopsy. In the Pediatric Surgery Department, the patient was advised to undergo surgery to remove the diseased tissue and reconstruct the area. However, the patient’s parents decided not to proceed with this step because the surgery would be very extensive, risky, and disfiguring, with an uncertain outcome. Removing all of the diseased bone is impossible, which means there is a risk that the lesion could change its dynamics and continue to grow. The girl is being treated conservatively, with an MRI with contrast performed approximately every six months. She is also receiving infusions of bisphosphonates. Unfortunately, this drug has not caused a regression of the lesion but is being administered to limit the progression of the disease. The most recent MRI, performed in January 2024, showed the lesion size as 6.0 × 4.2 × 6.2 cm. Fortunately, the patient does not experience the characteristic pain of dysplasia, although she has limited ability to breathe through her nose but functions normally. The patient remains under regular supervision at the Pediatric Outpatient Otolaryngology Clinic and with an ophthalmologist [11].

## 3. Discussion

**Figure 4:**
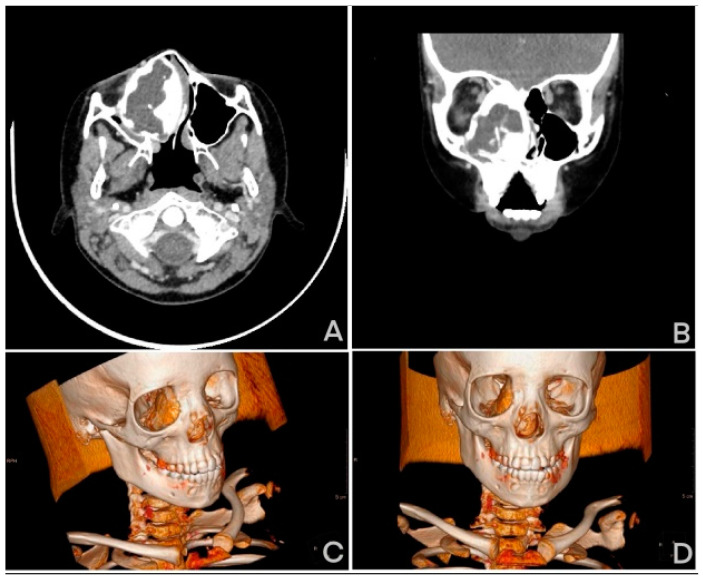
CT image in: (**A**) sagittal projection, (**B**) frontal, (**C**,**D**) 3D image reconstructions.

Diagnostic challenges in identifying fibrous dysplasia of bone arise from the rare location of the lesions in this anatomical region and the considerable variability in CT and MRI imaging, due to the varying proportions of fibrous and bony tissue within the affected bones [7]. The most commonly affected bones are the maxilla (12%) and mandible (12%), while the involvement of the ethmoid, sphenoid, frontal, and temporal bones is less frequent [6]. Due to advanced diagnostic tools, like endoscopy, computed tomography (CT), and magnetic resonance imaging (MRI), conditions affecting the ethmoid sinus are being identified more frequently [12]. Additionally, a complicating factor in diagnosis is the similarity of radiological images to many other bone conditions. Diagnostic errors are common in imaging studies (CT and MRI), where fibrous dysplasia is often mistaken for chondrosarcoma, sinusitis, meningioma, inflammatory polyps, adenoma, aspergilloma, and old fractures [7] (Figure 4). On the other hand, the literature describes the fibrous dysplasia involving craniofacial bones as the most frequently diagnosed form among all locations of fibrous dysplasia, due to its visually apparent manifestations, as observed by Adeyemo in 100% of cases [13]. However, this condition primarily affects the more visible craniofacial bones, such as the maxilla or mandible, where the manifestations are more noticeable. Early diagnosis of fibrous dysplasia in the ethmoid bone is infrequent at this stage, as it presents with nonspecific symptoms [3]. Although sinus involvement frequently has no symptoms, it can manifest as rhinitis, nasal congestion or obstruction, sinusitis, pain, or headache [4]. Furthermore, given that fibrous dysplasia is estimated to occur before 15 years of age, and its main development occurs during adolescence, the most common period for clinical manifestations and diagnosis is around 10 years of age [3].

Due to diagnostic uncertainties following a CT scan examination in our 10-year-old patient, it was concluded that histopathological evaluation is decisive in distinguishing fibrous dysplasia and serves as the basis for the final diagnosis. Localizing lesions within the ethmoid sinus necessitates a higher level of surgical expertise to obtain tissue samples. According to the international literature, an endoscopic biopsy approach was employed in the described scenario. This method offers a less invasive means of accessing areas that traditionally require a more invasive transcranial approach [7].

Currently, there is no established consensus on the treatment of craniofacial FD, and no therapy exists to limit the expansion of the disease [9]. Bisphosphonates are often used; however, they only contribute to pain relief [14,15]. Recent studies indicate that Denosumab (a RANKL inhibitor), may effectively control disease activity, but further research is needed to determine the potential benefits [16,17]. The preferred approach in managing craniofacial fibrous dysplasia involves symptomatic treatment, due to the benign nature of the condition [7]. Lesions in the naso-ethmoid region can result in airway compromise and displacement of the eyeball, leading to substantial deformity and functional impairment. It is advised to undergo an evaluation by an otorhinolaryngologist and a comprehensive ophthalmological assessment. Treatment approaches focus on alleviating airway obstruction, restoring normal globe position, visual function, and addressing physical deformities [3]. The patient should be advised to promptly report any changes in their vision, including loss of color perception and especially any sudden decline in vision, as these are absolute indications for therapeutic optic nerve decompression [18].

Surgical intervention is recommended only for patients with severe functional impairment, progressive deformities, or malignant transformation [7]. Surgical interventions in this region may involve partial or extensive excision with subsequent reconstruction of the skull base and orbits [3]. Conservative surgery may not be sufficient for all cases of craniofacial fibro-osseous lesions. For more extensive lesions, a more radical approach, such as craniofacial resection or total maxillectomy, may be necessary [18]. Radiation therapy is contraindicated due to the risk of malignant transformation. The surgical procedures do not negatively impact the growth rates of healthy tissue, and do not seem to trigger the onset of malignancy [18]. The risk of malignant transformation is estimated to be less than 1% for the monostotic form, and around 4% for the polyostotic form [7]. Patients with moderate to severe disease, particularly those experiencing significant functional disabilities or craniofacial deformities affecting appearance, should be considered for referral to a psychologist. Additionally, a referral to a social worker might also be necessary [7]. It is also advised to evaluate the psychological impact on both the patient and their family using the Craniofacial Experience Index [19].

## 4. Conclusions

Fibrous dysplasia is an uncommon bone disorder, very rarely occurring in children. Nevertheless, it is very important to consider it during diagnosis. There are both conservative and surgical therapies that can be applied. It is very important to provide the patient with psychological care.

## Data Availability

Data are contained within the article.
